# BNCT induced immunomodulatory effects contribute to mammary tumor inhibition

**DOI:** 10.1371/journal.pone.0222022

**Published:** 2019-09-03

**Authors:** Aslam Ali Khan, Charlie Maitz, Cai Quanyu, Fred Hawthorne

**Affiliations:** 1 International Institute of Nano and Molecular Medicine, University of Missouri, Columbia, United States of America; 2 Bond Life Science Center, University of Missouri, Columbia, United States of America; 3 Department of Veterinary Pathobiology, University of Missouri, Columbia, United States of America; Central Research Institute of Electric Power Industry (CRIEPI), JAPAN

## Abstract

In the United States, breast cancer is one of the most common and the second leading cause of cancer-related death in women. Treatment modalities for mammary tumor are surgical removal of the tumor tissue followed by either chemotherapy or radiotherapy or both. Radiation therapy is a whole body irradiation regimen that suppresses the immune system leaving hosts susceptible to infection or secondary tumors. Boron neutron capture therapy (BNCT) in that regard is more selective, the cells that are mostly affected are those that are loaded with 109 or more 10B atoms. Previously, we have described that liposomal encapsulation of boron-rich compounds such as TAC and MAC deliver a high payload to the tumor tissue when injected intravenously. Here we report that liposome-mediated boron delivery to the tumor is inversely proportional to the size of the murine mammary (EMT-6) tumors. The plausible reason for the inverse ratio of boron and EMT-6 tumor size is the necrosis in these tumors, which is more prominent in the large tumors. The large tumors also have receding blood vessels contributing further to poor boron delivery to these tumors. We next report that the presence of boron in blood is essential for the effects of BNCT on EMT-6 tumor inhibition as direct injection of boron-rich liposomes did not provide any added advantage in inhibition of EMT-6 tumor in BALB/c mice following irradiation despite having a significantly higher amount of boron in the tumor tissue. BNCT reaction in PBMCs resulted in the modification of these cells to anti-tumor phenotype. In this study, we report the immunomodulatory effects of BNCT when boron-rich compounds are delivered systemically.

## Introduction

Boron neutron capture therapy (BNCT) requires the selective delivery of boron-10 (^10^B) to tumor cells [1]. Following irradiation with neutrons, the nuclear capture and spontaneous fission reactions produce ^4^He and ^7^Li nuclei along with 2.4 MeV. These high-linear energy transfer (LET) particles travel less than ten micrometers from their sites of origin; therefore, they are only lethal to those cells that bind or internalize ^10^B in sufficient concentrations [2]. The effectiveness of BNCT is dependent upon the amount of ^10^B delivered per cell. BNCT has been used in the experimental treatment of a number of different tumors, such as Glioblastoma [3] [4] [5], skin melanomas [6], head and neck cancer *[7]*, mesothelioma [8], and diffuse liver metastases and could provide a useful treatment option for tumors that are unaffected by conventional therapies or that are difficult to remove surgically [9]. When metastases spread through an entire organ, the use of a selective BNCT agent might allow the selective destruction of each of the individual cells of the tumor nodules without requiring their selective irradiation (13). Selective ^10^B incorporation into cancer cells requires boron carrier molecules that exhibit a particular affinity toward the targeted cells [10]. A wide range of boron carriers has been designed, synthesized, and evaluated during the past several decades, including liposomes [11] [12]. Liposomes are slightly more advantageous for the selective delivery of ^10^B to murine EMT-6 tumors due to increased tumor cell growth rates and the incorporation of liposome components into the cellular membrane [13] [14] [15, 16]. Additionally, the often tortuous and leaky tumor vasculature allows the accumulation of boron within the tumor interstitium. Liposome delivery to the tumors depends upon the blood supply to the tumors, with a higher blood supply resulting in a higher ^10^B accumulation and lower blood supply culminating in lower boron accumulation in the tumors.

Macrophage/monocytes are professional phagocytes and phagocytize cell debris, foreign particles such as bacteria, fungi and parasites or liposomes and other particles of similar size. The phagocytosis of liposomes results in boron accumulation in macrophages/monocytes. Neutron irradiation may result in BNCT reaction and the energy released could modify macrophages to either tumor promoting or tumor inhibiting phenotype. Macrophage polarization is the hallmark of the innate immune response against cancer and pathogenic invasion. The extent of macrophage modification is dependent on the microenvironmental factors and decides the fate of macrophage polarization. The polarization of macrophages is multifaceted due to the plasticity of macrophages which can accommodate signals from pathogens, injured tissues, and the basal tissue microenvironment. The polarization of macrophages is under the control of pathways which regulate the survival of the cell by either prolonging or reducing macrophage development and viability. The tissue microenvironment, microbial products, and cytokines decide the fate of macrophage polarization. The activation of macrophages influences other branches of the immune system due to these cells being the essential modulators and effectors of the immune response. A hypothesis put forward that subsets of T helper cells can be distinguished based on the cytokines secreted after their activation. These subsets mediate distinct regulatory and effector functions [17]. In 1960s Mackaness introduced the term macrophage activation (classical activation) about infection to describe the antigen-dependent but non-specific anti-microbial response of macrophages to BCG (bacillus Calmette-Guerin), and Listeria upon subsequent exposure [18–29]. Later the enhanced microbicidal activity was linked to T helper type 1 responses, along with IFNγ release by antigen-activated immune cells [[30]] and these microbicidal effects regulated by TH1 and IFNγ also account for cytotoxic and antitumoral effects ([31, 32]). Stein, Doyle, and colleagues postulated that IL-4 and IL-13 induces an alternative activation phenotype due to the discovery of the mannose receptor which selectively enhances the TH2 response in murine macrophages. The alternative activation is an entirely different phase than classical activation, but different from deactivation. In other words, the lack of a classical activation does not entail loss of activation [33, 34].

Macrophages in mice with TH1 and TH2 backgrounds differed in the propensity to react to the classic stimuli (IFNγ or lipopolysaccharide or both). The ability to respond to stimuli carves a vital difference in metabolic pathways in macrophages. The macrophage nomenclature of M1 and M2 is similar and based on the activation of the T helper type cells, i.e., the macrophages which activate TH1 cells belong to the M1 phenotype, while M2 macrophages activate TH2 cells. For example, M1 macrophages following LPS or IFN stimuli release toxic nitric oxide (NO), while M2 macrophages release polyamines ([35, 36]). The official report for the alternative activation of macrophages in vivo similar to the observation of Mackness for pathogens came from the observation of Allen, de Baetselier, Brombacher, and colleagues in parasite infection. The parasite elicits a strong IgE and TH2 response [[37]]. Montavani and colleagues grouped the macrophage activating factors in two functionally polarized states to integrate the phenotypic similarities and differences. The macrophages were grouped based on their effects on select markers as M1 (IFNγ and LPS or TNF-α), M2a (IL-4), M2c {IL-10 and glucocorticoids (GCs).

## Results

### Optimizing the boron delivery to the EMT-6 tumors for effective boron neutron capture therapy (BNCT)

**Fig 1:**
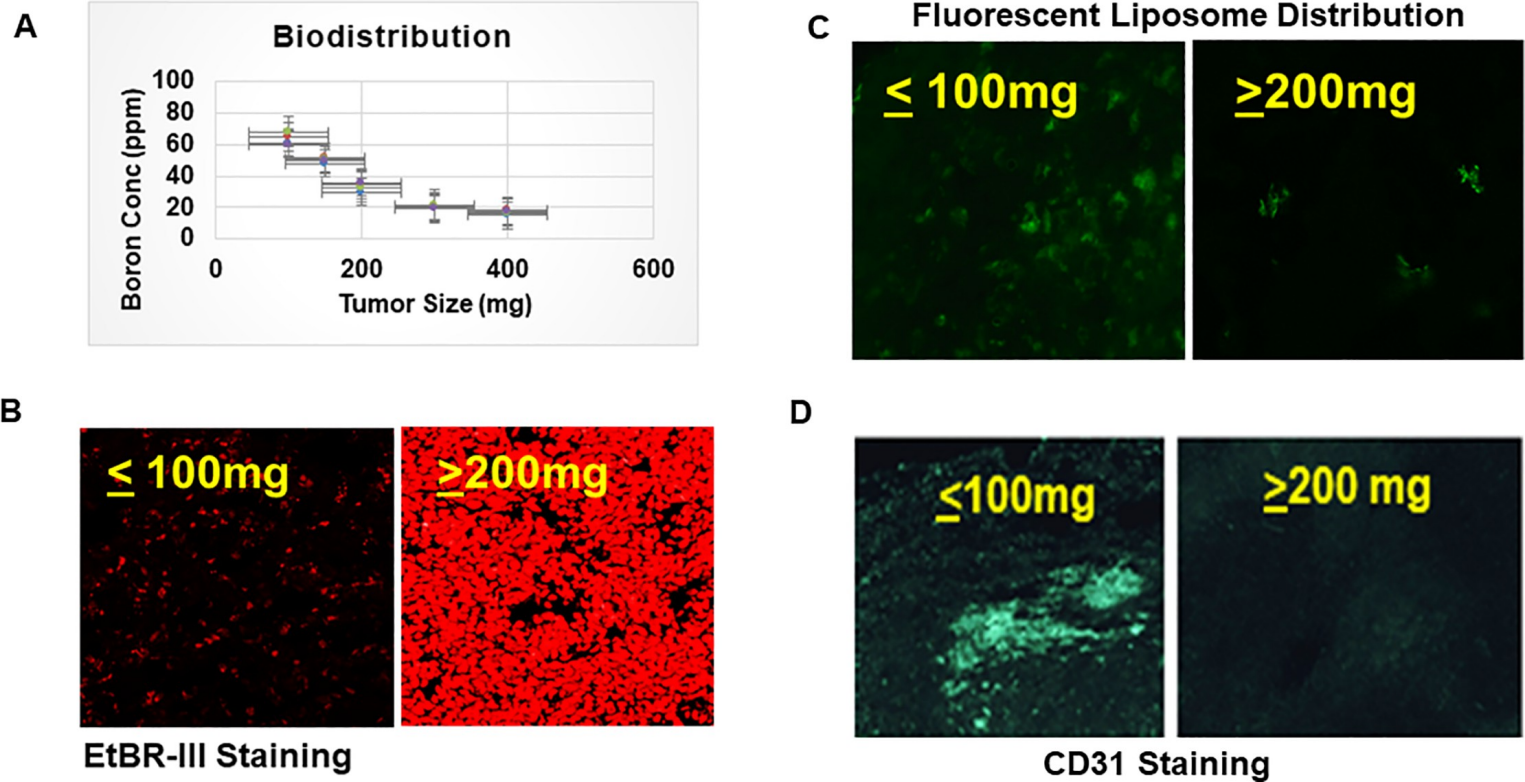
Higher accumulation of boron in small tumors than large tumors due to necrosis and receding blood vessels. **A.** EMT-6 cells, at a density of 1X10^6^ cells per mouse in BALB/c mice (n = 5/set), were injected to induce tumors (100–400 mg) followed by injection of TAC and MAC-containing liposomes for different time points (18–72 hrs). The tumors were harvested and blood was collected in heparinized tubes to prevent clotting and contents were determined by ICP-OES. **B.** BALB/c mice (n = 5/set) received EMT-6 cells, at a density of 1X10^6^ cells per mouse, in the right flank. Mice were euthanized at different times to harvest tumors (100-400mg). Harvested tumors were fixed and cryoprotected using 4% paraformaldehyde and 30% sucrose in PBS. Next, 10-μm sections were cut using a cryostat and stained with EtBRIII to evaluate necrosis. **C.** BALB/c mice (n = 5/set) received EMT-6 cells in their right flank at a density of 1X10^6^ cells per mouse. Mice were injected with fluorescent MAC-TAC liposomes for optimal time point of 54 hrs before euthanization at different times to harvest the tumors of different sizes (100-400mg). The tumors were then fixed and cryoprotected using 4% paraformaldehyde and 30% sucrose in PBS. Next, 10-μm sections were cut using a cryostat and observed under the fluorescent microscope and picture taken. **D.** BALB/c mice (n = 5/set) received EMT-6 cells in their right flank at a density of 1X10^6^ cells per mouse. Mice were euthanized at different times to harvest the tumors of different sizes (100-400mg). The tumors were then fixed and cryoprotected using 4% paraformaldehyde and 30% sucrose in PBS. Next, 10-μm sections were cut using a cryostat and stained with anti-CD31 antibody labeled with FITC for blood vessel analysis. Statistics student unpaired t-Test was used to determine the significance between each point and groups. * = P value of ≤ 0.05, ** = P value of ≤ 0.01, *** = P value of ≤ 0.001.

We evaluated the boron accumulation in EMT-6 tumors of different sizes and observed that boron accumulation was extremely low in larger tumors (approximately 400 mg) but was significantly higher in smaller tumors (approximately 100 mg) (Fig 1A). These results suggest smaller EMT-6 tumors are more amenable to boron delivery. We further tested the boron distribution by incorporating fluorescein dye in liposome in order to track the boron distribution in tumors of varying size. distribution. We observed the uniform distribution of fluorescence in small tumors while fluorescence in large tumors was spotty and did not cover the large tumor area. (Fig 1B). We evaluated the necrosis present in EMT-6 tumors ranging from 100–400 mg in size. Various 10-μm cryosections of the EMT-6 tumors revealed that necrosis progressively increases with the size of the tumor (Fig 1C). These regions represent oxygen deprivation areas [38] [39] and [40] hence cell death by mostly necrosis.

Blood vessels are vital for the growth and development of tumors and the delivery of nutrients and drugs. As we have shown above, boron distribution is affected by tumor size. We examined tumor vasculature by staining tumor sections with an anti-CD31 antibody labeled with Fluorescein Isothiocyanate (FITC). CD31 staining of the EMT-6 tumor sections revealed that smaller EMT-6 tumors have a higher amount of CD31 staining than the larger EMT-6 tumors (Fig 1D).

### Immunomodulatory effect of BNCT in EMT-6 tumors

**Fig 2:**
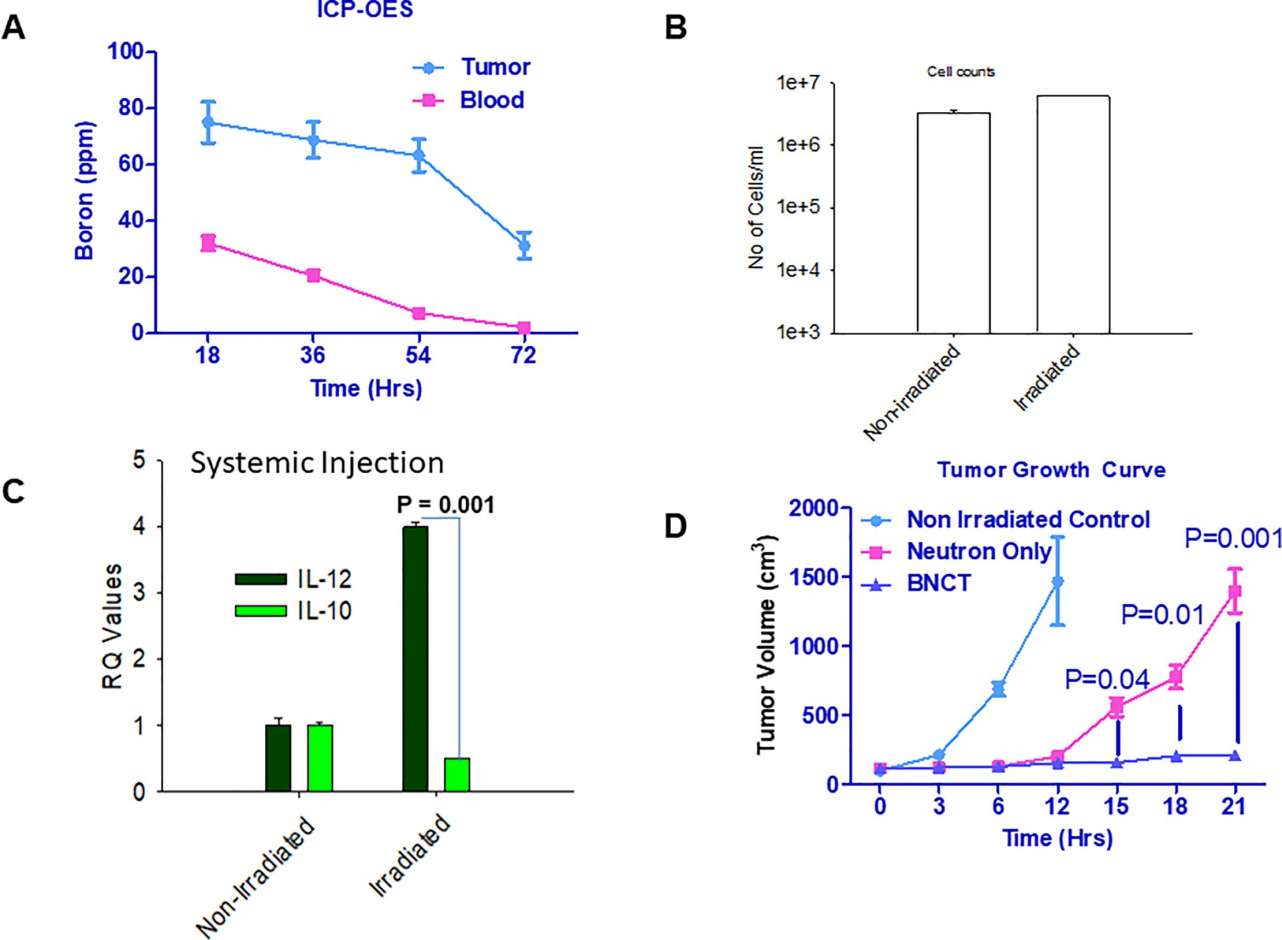
Systemic delivery of boron-rich liposomes affects tumor growth via modulation of PBMCs. ***A*.** EMT-6 (n = 5/set) tumor-bearing BALB/c mice were injected with TAC/MAC liposomes for different time points. Mice were euthanized followed by the collection of tumors and blood from these mice. Boron levels in tumor and blood were analyzed using ICP-ES. ***B*.** BALB/c mice (n = 5/set) were implanted with EMT-6 cells in the right flank. The mice were irradiated using a neutron beam for 30–45 mins followed by resting of the mice for 24 hrs to reduce the radioactivity in mice occurring due to irradiation. Blood was collected from irradiated and non-irradiated mice in EDTA-containing tubes to prevent clotting. Blood was diluted with 1XPBS 10 times and layered onto histopaque to isolate PBMCs by density centrifugation. The isolated cells were washed and mixed with trypan blue to assess the viability using an automatic cell counter. ***C*.** The PBMCs collected in Fig 2B were lysed using RNA lysis buffer provided with RNA isolation kit and RNA isolated. cDNA was prepared using cDNA kit followed the analysis for IL-12 and IL-10 using real-time PCR probes. ***D*.** EMT-6 tumor-bearing BALB/c mice (n = 5/set) were injected with TAC/MAC liposomes for 54 hrs followed by irradiation of mice for 30–45 mins. Tumors were allowed to grow in mice following irradiation till the tumors in neutron only irradiated mice reached to one gram. Statistics student unpaired t-Test was used to determine the significance between each point and groups. * = P value of ≤ 0.05, ** = P value of ≤ 0.01, *** = P value of ≤ 0.001.

After optimizing the delivery of boron compounds in tumor tissue, we then moved to irradiation studies. One set of EMT-6 tumor laden mice received neutron irradiation, and the second set did not receive radiation but only the liposomes for biodistribution. Time kinetics of of boron distribution in tumor and blood revealed that tumors had approximately 60 ppm of boron and blood had 10 ppm of boron (Fig 2A). The optimal tumor/blood ratio and optimal boron concentration in tumors was observed 54 hrs post systemic delivery of boron-rich liposomes. We used 54 hrs as optimal time point for optimal boron dose in all our irradiation experiments. We used this time point for all the irradiation studies. In the blood, peripheral blood mononuclear cells (PBMCs) are most likely to assimilate liposomes and hence boron, than other components of the blood.

Analysis of blood following irradiation revealed that irradiation did not cause damage to the PBMCs despite these cells carrying boron which is why the total population of PBMCs remain unchanged (Fig 2B). IL-12 is a known anti-tumor cytokine and also protect against infection caused by invading pathogens. In tumor microenvironment the intruding macrophages are modified to suppress IL-12 expression and enhance IL-10 expression. IL-10 suppresses anti-tumor immune response by modifying macrophages to release tumor promoting factors. Analysis of expression of IL-12 and IL-10 suggested that the PBMCs switched their phenotype to anti-tumor phenotype with increased levels of interleukin-12 (IL-12) and decreased IL-10 levels (Fig 2C). We observed that irradiation of EMT-6 tumor-bearing mice significantly inhibited tumor growth in these mice. The point of significance in the tumor growth inhibition started at around 15 days (P = 0.04) and kept on increasing till the time study was ended at 22 days (P = 0.001) due to large tumor sizes and discomfort and restricted movement of neutron only irradiated mice (Fig 2D).

**Fig 3:**
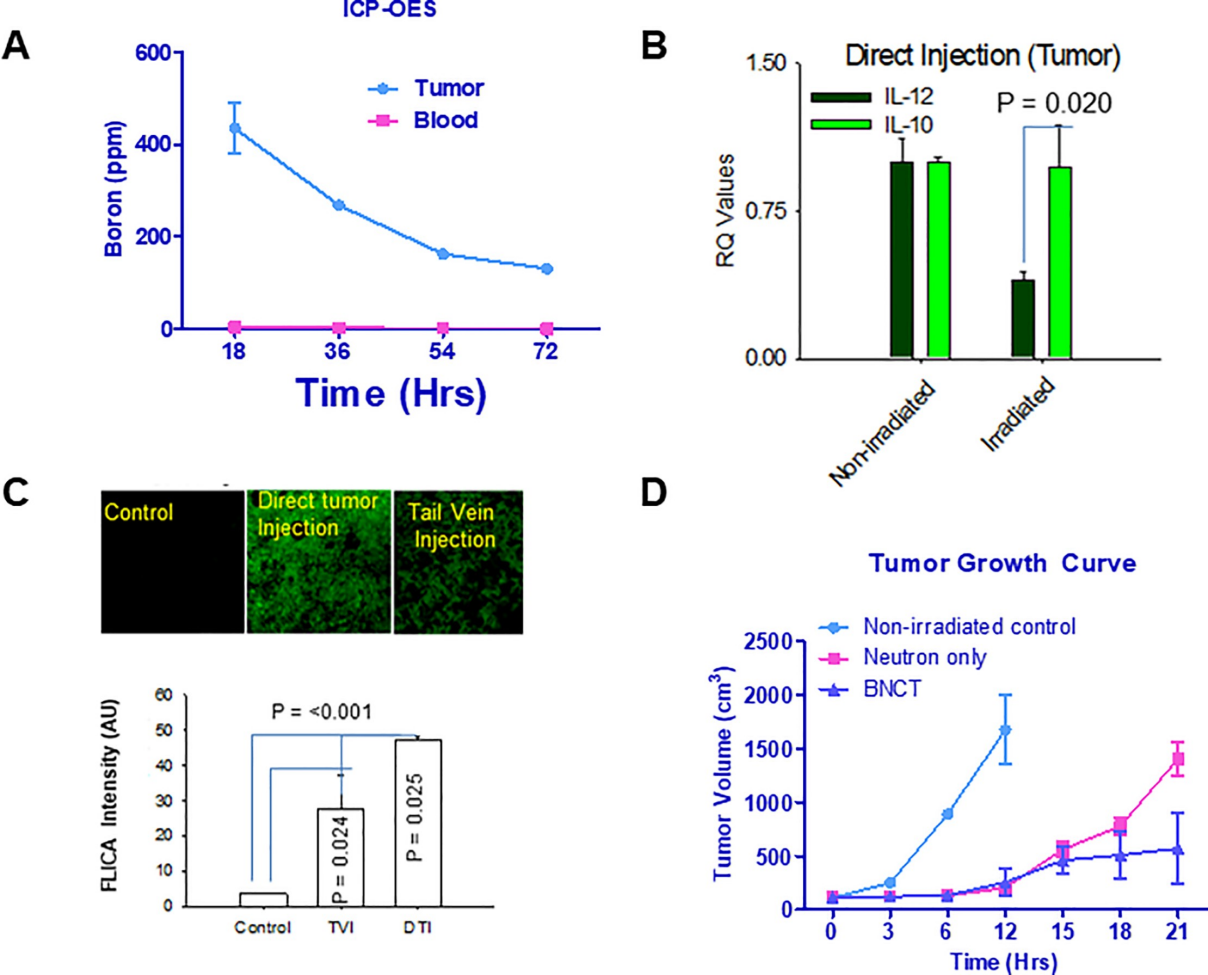
Direct tumor injection of TAC/MAC liposomes did not affect the cytokine profile and tumor growth as compared to neutron only irradiation. ***A*.** EMT-6 (n = 5/set) tumor-bearing mice received the direct tumoral injection of TAC/MAC liposomes for indicated time points followed by collection of blood and tumors. The blood and tumor tissues were analyzed for boron by ICP = OES. **B.**BALB/c mice (n = 5/set) were impanted with EMT-6 cells and blood was collected following irradiation of mice. The PBMCs were analysed for IL2 and IL-10 cytokine levels by RT-PCR. ***C*.** Tumors isolated from above mice were fixed and cryoprotected using 4% paraformaldehyde and 30% sucrose in PBS. Next, 10-μm sections were cut using a cryostat and stained with FLICA and pictures taken under a fluorescence microscope. ***D*.** BALB/c mice (n = 5) were implanted with EMT-6 cells in the right flank. Mice were injected with TAC/MAC liposomes directly into the tumor followed by neutron irradiation. The tumors were then allowed to grow in mice till the control tumors (neutron only) reached the size of 1gram or mice had problems in walking or deterioration of overall health. Statistics: Unpaired student t-Test was used to determine the significance between each point and groups. * = P value of ≤ 0.05, ** = P value of ≤ 0.01, *** = P value of ≤ 0.001.

Time kinetics of boron distribution following direct tumor injection of boron-rich lipososmes suggested that the boron concetration did not decrease substantially (Fig 3A). Direct injection of boron-rich liposomes into tumor did not have any effect on PBMCs cytokine profile following irradiation which remained as low IL-12 and high IL-10 levels (Fig 3B). Caspases play important roles in the induction of cells. The caspase staining with FLICA (Fluorescent inhibitor of Caspase) to assess apoptosis following irradiation revealed that the tumors injected directly with boron-rich liposomes has significantly elevated caspase activity than the systemic delivery of boron-rich liposomes to the tumors (Fig 3C). Irradiation of tumor-bearing mice following direct injection of boron-rich liposomes did not show increased inhibition of EMT-6 tumor growth despite the presence of boron at more than double the concentration compared to intravenous delivery (Fig 3D). Infact the inhibition of tumor growth did not reach the point of significance.

## Discussion

Selective delivery of boron forms the basis of effective BNCT for the treatment of solid tumors [41] [1]. The capture of low-energy neutrons by ^10^B results in fission reactions leading to the generation of high energy He2+ and Li3+ particles which traverse the path of 5–10 μm in diameter from initial point and hence are lethal to the cells carrying boron in sufficient concentrations. The results of our study indicate that boron distribution in the tumors is critical for effective BNCT therapy. We have shown that the size of the tumor is inversely proportional to the accumulation of boron compounds. We have further demonstrated that the reasons for low boron distribution include the high levels of necrosis and the fewer number of blood vessels observed in larger EMT-6 tumors. In other words, vascularization of EMT-6 tumors is inversely proportional to the necrosis in these tumors. Further vascularization of EMT-6 tumors directly proportional to the distribution of boron rich liposomes in these tumors and thus affect the efficiency of BNCT in treating the mammary tumors. Our results further reveal that boron-rich liposomes assimilated by PBMCs activated and modified these cells to an anti-tumor phenotype and aided in the inhibition of tumor growth. We observed that the tumor growth following systemic or direct tumor delivery of boron-rich liposomes revealed that the initial effects of irradiation in terms of caspase activity was higher in tumors directly injected with boron-rich liposomes than tumors receiving boron-rich liposomes systemically, however the long term effect on tumor growth were significantly higher in tumors receiving boron-rich liposomes systemically. These results suggest that vascular and liposomal delivery of boron compounds might have immunomodulatory effects and hence overtakes the overall impact on tumor growth. Eventhough the initial caspase activity is higher in tumor sections and cells obtained from the mice receiving direct tumoral injecrtion of boron rich liposomes, but overall tumor inhibition is lower than in the mice receiving systemic delivery. The plausible reason for the differential effect could be that intra-tumoral boron delivery produces acute effects while immune system modulation could produce long lasting effect. Similar, plausible immunomodulatory effects were seen in a study where rats implanted with colon tumors in both right and left legs followed by exposure to neutron beam in one leg resulted in significant reduction of tumors non-exposed leg [42]. In this study boronophenylalanine (BPA) as a source of boron for BNCT reaction [42]. Furthermore, cancer cells damaged by irradiation can also undergo autophagy to chew-up the damaged part to prevent cell death [43]. In this study, we are showing initial results where boron-rich liposomes can modify the PBMCs to antitumor phenotype.

Radiation therapy is the conventional treatment modality along with chemotherapy following surgical removal of tumor tissue to prevent relapse perpetrated by residual tumor tissue or cells. These therapies, however also result in severe side effects in patients thereby affecting their quality of life. There are two forms of radiation therapy, one is whole body irradiation therapy or radiation of exposed part making it somewhat taregeted, however the radiation does not differentiate between cancerous or non-cancerous tissue or cells, and the other is a specific therapy where radiation only affects the cells carrying the target molecule. In our study we observed little to no boron distribution in tissues such as heart, brain, kidney and lungs thereby preventing off target effects. We did not see the irradiation effects on noncancerous tissue probalby due to low boron distribution and hence proving our initial point of BNCT being a more targeted therapy than other forms of irradiation. The immunomodulatory effects occurring due to systemic delivery of boron molecules needs to be studied comprehensively. We are sharing our initial observation here for the scientific community so that it can ponder upon the therapeutic effects of BNCT before writing it off as another means of radiation therapy. BNCT has the potential to become an essential form of treatment for various cancers. However, most of the compounds developed for use in BNCT generally do not demonstrate significant inhibition of tumor growth, which could be the result of poor distribution of boron in tumors and also the mode of delivery. We suggest that for BNCT to be an effective therapy for inhibition of tumor growth or prevention of relapse in patients, the delivery of boron-rich compounds should be systemic and by nano drug delivery systems. The nano-delivery systems will deliver boron to the PBMCs which might activate these cells and help in the elimination of tumors.

## Materials and methods

### Animals

All work was performed by the general protocols of animal care, and experimental design committee and the experiments outlined in this study are on file and have been approved by the University of Missouri Committee on the Humane Care of Laboratory Animals (CHCLA). All animal caretakers and laboratory personnel have appropriate approvals based on specific American Association for Laboratory Animal Care (AALAC)-approved training programs. The facilities are regularly inspected by University Committee and by unannounced visits directed by the Federal Government. We obtained BALB/c mice from Harlan Sprague (Indianapolis, IN). Minimum of 10 mice were used in all the mice experiments listed in this study. All the animal experiments were done according the ACUC protocol # 7993.

### Reagents, and cell lines

We purchased Distearoyl-*sn*-glycero-3-phosphocholine (DSPC) from Avanti Polar Lipids (Alabaster, AL), Cholesterol from Sigma-Aldrich (St. Louis, MO) and syringe filters from Corning (Lowell, MA). The Sephadex G-25 gel used in the study was purchased from Sigma-Aldrich (St. Louis, MO). We bought the anti-CD31 FITC antibody from Thermo Fisher Scientific (Rockford, IL), the tissue-freezing medium from the Molecular Cytology Core Facility (University of Missouri, Columbia, MO), the EtBRIII (necrosis study) from Enzo Life Sciences (Farmingdale, NY). Lastly, the EMT-6 cells from ATCC (Manassas, VA).

### MAC/TAC liposome preparation

First, 0.1675 g of 1,2-distearoyl-*sn*-glycero-3-phosphocholine (DSPC) was added to a 16 x 100 mm glass tube. Next, 0.0820 g of cholesterol (Sigma-Aldrich, St. Louis, MO) and 0.0505 g K[*nido*-7-CH_3_(CH_2_)_15_−7,8-C_2_B_9_H_ll_] (MAC) was added to achieve 0.3 g total of a 1:1:0.6 molar ratio of the respective components. The mixture was dissolved in chloroform and methanol before being vortexed, dried by a nitrogen stream until a film formed and then further dried for 12 hrs in a vacuum. Next, 6 ml of sterile, aqueous 250 mM (1000 mOsM) Na_3_ [1-(2'-B_10_H_9_)-2-NH_3_B_10_H_8_] (TAC), which had been adjusted to pH seven via the addition of 1 M HCl, was added to the vacuum-dried lipid mixture. The mixture was vortexed then sonicated for 30 minutes at 65°C using a Sonics & Materials Vibracell and a 1/8” standard tapered microtip probe. Following sonication, the liposome mixture was purified from unincorporated materials via size-exclusion chromatography using Sephadex G-25 (medium) filtration with phosphate buffered lactose (9% w/v lactose, five mM phosphate, pH 7.4) as the eluting buffer solution. The collected and combined liposome suspension was then filtered and sterilized by passage through two 0.2 μm Corning syringe filters directly into an autoclave-sterilized, sealed serum bottle. Particle sizing was achieved utilizing a Zetatrac particle analyzer from Microtrac, Inc. (Montgomeryville, PA) [44, 45].

### Cell culture, tumor induction, and experimental design

We obtained EMT6 cells from American Type Culture Collection (ATCC) and cultured in DMEM medium supplemented with 10% FBS as recommended by ATCC. TrypLE buffer (Life Technologies) was used to dissociate cells in log phase, DMEM + 10% FBS was added to stop TrypLE reaction. The cells were pelleted down using accuspin 3R centrifuge (Fisher Scientific) at 323 × *g* for 8 min at room temperature, followed by resuspension of cell pellet in 1X phosphate buffered saline (PBS). Cells number was counted using an Automatic Cell Counter (Life Technologies). For tumor induction, one million EMT6 cells (1 × 10^**6**^ cells/mouse) per mouse were inoculated into female BALB/c (n = 5/set) mice’s right flank. The mice were typically weighed around 20 ± 1 g for all the studies listed in this manuscript as described previously [11].

### Quantitative analysis of boron distribution in mice tissues using ICP-OES

BALB/c mice (n = 5/time point) were implanted with EMT-6 tumors in the right flank of these mice. When the tumor size reached the desired size, the mice were injected with TAC/MAC liposomes either intravenous or directly into tumors for indicated time points. The injected dose of liposomes contained the boron at concentration of 350 μg or 17.5 μg 10B/gram of body mass. The mice were then euthanized by first anaesthetizing with cocktail of 10 mg/kg xylazine and 80 mg/kg ketamine followed by cervical dislocation. We collected tissue samples (blood and tumor) in clean, and dry tubes following intravenous or direct injections. Optima-grade nitric acid (1 ml) was added to the blood and tumor samples and allowed to sit followed by shaking. Also, 100 μl of scandium (200μg/ml) was added to each pressure vessel to serve as a standard for all samples. We used CEM Mar microwave for tissue digestion followed by measurement of tube weight after sample dilution. The samples were transferred to appropriately labeled 50-mL conical tubes and analyzed by ICP. Plasma was used as the source of light to excite the samples. The light intensities of calibrants were used to calculate the sample boron concentration. The calibrants (7 ml each) and a blank were placed in the auto-sampler and analyzed. We used a blank between each sample analysis, and a spiked solution was used approximately every four samples to ensure proper instrument function.

### Time kinetics for biodistribution studies

The biodistribution studies BALB/c mice (n = 5/set) were implanted with EMT-6 tumor cells. The mice were injected with boron-rich liposomes for different time points either systemically or direct tumor injections. The mice euthanized and tumors and blood were harvested and analyzed for boron conc by ICP-OES. Boron levels in tumor tissue following systemic delivery of boron-rich liposomes peaked in 54 h. At 54 h, boron concentration in the tumors was 67.8 μg ^10^B per gram tumor, and the tumor/blood boron ratio was 10:1. As clearance of boron from blood proceeded more rapidly than loss from tumors, the tumor/blood ratio continued to increase till 54 hrs. We used 54 hrs as an optimum time point for irradiation.

### Study of tumor necrosis

The BALB/c mice (n = 5/set) were injected with EMT-6 cells at a density of 1X10^6^ per mouse and allowed to grow to tumors of different sizes (80 mg-400 mg). Tumors harvested following euthanization as described above. The tumors were then incubated overnight in 4% paraformaldehyde and then washed with 1X PBS. The tumors were incubated in a 30% sucrose solution at 4°C overnight before being washed in a solution of 30% sucrose in distilled water. The tumors were then frozen in tissue-freezing medium (TFM) and sectioned in 10-μm-thick slices. The sections were then stained with EtBRIII and observed using a confocal microscope at a wavelength of 600 nm, and images taken of the sections.

### CD31 staining of the tumor section

BALB/c mice (n = 5/set) were implanted with EMT-6 tumor cells in the right flank. Tumors were allowed to grow to indicated sizes and sectioned as described above and previously [46]. Briefly, the tumor sections were stained with anti-mouse CD31-FITC overnight at 4°C. The sections were then washed with 1X PBS and observed under a confocal microscope at an excitation wavelength of 488 nm, and images taken of the sections [47].

### Neutron irradiation

BALB/c mice (n = 5/set) were implanted with EMT-6 cells in the right flank. When tumors were 80- to the 150-mm^3^ in volume, ^10^B-enriched boronated liposome suspensions were administered to the mice either via tail-vein or direct tumor injections. The mice were administered intraperitoneally (i.p.) with cocktails of 10mg/kg xylazine and 80mg/ml of ketamine, and Cu/Au flux wires were implanted on the left and right thorax of each mouse before irradiation. ^6^LiCO_3_ was used to shield the head, thorax, and cranial abdomen of the mice during irradiation to avoid possible complications which also helped in improving the specificity of the treatment. Once the mice were placed in positioning gallantry, the gallantry was introduced in the irradiation chamber and irradiated for 30–45 mins. A camera was set to monitor the discomfort in the mice during irradiation. Mice were taken out of the irradiation chamber and allowed to recover from anesthesia followed by a collection of the Cu/Au wires from the irradiated mice. The dose of irradiation was calculated as described ealeir [44, 45]

### Therapeutic effect studies

The effect of BNCT was determined based on tumor volume changes during the course of the study. The control group was not administered with the boron while receiving the same amount of irradiation as the boron group. Calipers were used to measure the tumor volumes during the course of the study. Mice were euthanized when tumor volume reached 2000 mm^**3**^, or the mice showed visible discomfort in moving and accessing the food and water *[44, 45]*. For effective BNCT mediated effects on tumors the total thermal (0–0.414 eV) neutron fluence must be at least 1 × 10^**12**^ neutrons/cm^**2**^. A 30–45 min exposure to the University of Missouri Research Reactor neutron beam provides a total thermal neutron fluence of 1.6–2,4 × 10^**12**^ which was sufficient to produce desired therapeutic effects on EMT-6 tumors. Boron dose and irradiation dose are calculated in Table 1.

**Table 1:** 

Initial injected dose	Observed dose (Tumor)	Irradiation dose (Gy) (Tumor)	Time of Irradiation (mins)
17.6 ug ^10^B/gram body mass	3μg/g body mass	7.75	30
35.2 μg ^10^B/gram body mass	6μg/g body mass	15.50	30
17.6 μg ^10^B/gram body mass	3μg/g body mass	11.625	45
35.2 ^10^B/gram body mass	6μg/g body mass	23.250	45
8.8 ug ^10^B/gram body mass	3μg/g body mass	3.875	30
8.8 ug ^10^B/gram body mass	3μg/g body mass	5.8125	45

***Initial Injected dose***- Dose of boron injected in the mice, ***Observed dose-***Boron amount in tumors as measured by ICP-OES (3 μg/gram = 3ppm (parts per million) for the mice of 20 gram makes it 60 ppm) ***Irradiation dose-*** Absorbed physical dose for boron capture was 12.9 cGy/ppm in tissues, so for 60 ppm of boron in tumor tissue the physical or irradiation dose was 12.9X60 = 774 cGy or ∼ 7.75 Gy.

### PBMCs count

The BALB/c mice (n = 5) injected with TAC/MAC liposomes for 48 hrs were either irradiated or left non-irradiated. Followed by a collection of blood through the heart puncture in a heparinized tube to prevent blood clotting. We isolated PBMCs by gradient centrifugation on Histopaque (Sigma, St Louis, MO) solution. The automatic cell counter was used to determine the PMBCs in blood collected from both irradiated and non-irradiated mice.

### Quantitation of cytokines in PBMCs following irradiation

Total RNA was prepared from PBMCs isolated from above mice using the RNeasy mini kit (Qiagen). cDNA was made using the High Capacity cDNA Kit (Applied Biosystems), and PCR amplification of cDNA was performed using the Taqman probe-based gene expression assay (Applied Biosystems) as previously described. The probes for IL-12 (Mm01288989_m1) and IL-10 (Mm00439614_m1) were used to determine the PBMCs polarization status [48].

### Caspase activity in tumors following irradiation

EMT-6 tumors were grown in four sets of BALB/c mice (n = 5/set) which either received direct tumor injection or tail vein injection for otpimum time point of 54 hrs when tumors reached the size of 100-150mg. Boron distribution in tumor and blood was done by ICP-OES in two sets of mice (direct tumor or tail vein injection). Other two sets (direct tumor tail vein injection) were irradiated as described previously. The tumors were then incubated overnight in 4% paraformaldehyde and then washed with 1X PBS, followed by incubation in a 30% sucrose solution at 4°C overnight before being washed in a solution of 30% sucrose in distilled water. The tumors were then frozen in tissue-freezing medium (TFM) and sectioned into 10-μm-thick slices. The sections were then stained with EtBRIII and observed using a confocal microscope at a wavelength of 600 nm, and images taken of the sections [49].

## References

[1] Yanagie H, Kumada H, Sakurai Y, Nakamura T, Furuya Y, Sugiyama H, et al Dosimetric evaluation of neutron capture therapy for local advanced breast cancer. Appl Radiat Isot. 2009;67(7–8 Suppl):S63–6. Epub 2009/05/12. S0969-8043(09)00248-6 [pii] 10.1016/j.apradiso.2009.03.110 . 19427224

[2] Backer MV, Gaynutdinov TI, Patel V, Bandyopadhyaya AK, Thirumamagal BT, Tjarks W, et al Vascular endothelial growth factor selectively targets boronated dendrimers to tumor vasculature. Mol Cancer Ther. 2005;4(9):1423–9. 10.1158/1535-7163.MCT-05-0161 . 16170035

[3] Joensuu H, Kankaanranta L, Seppala T, Auterinen I, Kallio M, Kulvik M, et al Boron neutron capture therapy of brain tumors: clinical trials at the finnish facility using boronophenylalanine. J Neurooncol. 2003;62(1–2):123–34. . 12749708 10.1007/BF02699939

[4] Blue TE, Yanch JC. Accelerator-based epithermal neutron sources for boron neutron capture therapy of brain tumors. J Neurooncol. 2003;62(1–2):19–31. . 12749700 10.1007/BF02699931

[5] Gupta N, Gahbauer RA, Blue TE, Albertson B. Common challenges and problems in clinical trials of boron neutron capture therapy of brain tumors. J Neurooncol. 2003;62(1–2):197–210. Epub 2003/05/17. . 12749714 10.1007/BF02699945

[6] Gonzalez SJ, Bonomi MR, Santa Cruz GA, Blaumann HR, Calzetta Larrieu OA, Menendez P, et al First BNCT treatment of a skin melanoma in Argentina: dosimetric analysis and clinical outcome. Appl Radiat Isot. 2004;61(5):1101–5. 10.1016/j.apradiso.2004.05.060 . 15308199

[7] Kankaanranta L, Seppala T, Koivunoro H, Saarilahti K, Atula T, Collan J, et al Boron Neutron Capture Therapy in the Treatment of Locally Recurred Head-and-Neck Cancer: Final Analysis of a Phase I/II Trial. Int J Radiat Oncol Biol Phys. 2011 Epub 2011/02/09. S0360-3016(10)03463-2 [pii] 10.1016/j.ijrobp.2010.09.057 . 21300462

[8] Suzuki M, Sakurai Y, Masunaga S, Kinashi Y, Nagata K, Maruhashi A, et al Feasibility of boron neutron capture therapy (BNCT) for malignant pleural mesothelioma from a viewpoint of dose distribution analysis. Int J Radiat Oncol Biol Phys. 2006;66(5):1584–9. 10.1016/j.ijrobp.2006.08.026 . 17056195

[9] Fujii H, Matsuyama A, Komoda H, Sasai M, Suzuki M, Asano T, et al Cationized gelatin-HVJ envelope with sodium borocaptate improved the BNCT efficacy for liver tumors in vivo. Radiat Oncol. 2011;6:8 10.1186/1748-717X-6-8 21247507 PMC3035588

[10] Wu G, Barth RF, Yang W, Lee RJ, Tjarks W, Backer MV, et al Boron containing macromolecules and nanovehicles as delivery agents for neutron capture therapy. Anticancer Agents Med Chem. 2006;6(2):167–84. . 16529539 10.2174/187152006776119153

[11] Shelly K, Feakes DA, Hawthorne MF, Schmidt PG, Krisch TA, Bauer WF. Model studies directed toward the boron neutron-capture therapy of cancer: boron delivery to murine tumors with liposomes. Proc Natl Acad Sci U S A. 1992;89(19):9039–43. Epub 1992/10/01. 10.1073/pnas.89.19.9039 1409600 PMC50060

[12] Feakes DA, Shelly K, Hawthorne MF. Selective boron delivery to murine tumors by lipophilic species incorporated in the membranes of unilamellar liposomes. Proc Natl Acad Sci U S A. 1995;92(5):1367–70. 10.1073/pnas.92.5.1367 7877984 PMC42520

[13] Thirumamagal BT, Zhao XB, Bandyopadhyaya AK, Naranyanasamy S, Johnsamuel J, Tiwari R, et al Receptor-targeted liposomal delivery of boron-containing cholesterol mimics for boron neutron capture therapy (BNCT). Bioconjug Chem. 2006;17(5):1141–50. 10.1021/bc060075d . 16984121

[14] Hawthorne MF, Shelly K. Liposomes as drug delivery vehicles for boron agents. J Neurooncol. 1997;33(1–2):53–8. . 9151223 10.1023/a:1005713113990

[15] Kang W, Svirskis D, Sarojini V, McGregor AL, Bevitt J, Wu Z. Cyclic-RGDyC functionalized liposomes for dual-targeting of tumor vasculature and cancer cells in glioblastoma: An in vitro boron neutron capture therapy study. Oncotarget. 2017;8(22):36614–27. 10.18632/oncotarget.16625 28402271 PMC5482681

[16] Nakamura H. Boron lipid-based liposomal boron delivery system for neutron capture therapy: recent development and future perspective. Future Med Chem. 2013;5(6):715–30. 10.4155/fmc.13.48 . 23617433

[17] Coffman RL. Origins of the T(H)1-T(H)2 model: a personal perspective. Nat Immunol. 2006;7(6):539–41. 10.1038/ni0606-539 . 16715060

[18] Shilpi RY, Sachdeva R, Simm M. Cellular resistance to HIV-1 infection in target cells coincides with a rapid induction of X-DING-CD4 mRNA: indication of the unique host innate response to virus regulated through function of the X-DING-CD4 gene. Innate Immun. 2012;18(4):563–70. 10.1177/1753425911426893 22042911 PMC3793846

[19] Schmitz B, Possart-Schmitz P, Gehrung M, Stauffer U, Mossmann H, Fischer H. Cellular response and resistance to the primary infection of rats and mice with Nematospiroides dubius. Z Parasitenkd. 1982;68(3):339–47. . 7157944 10.1007/BF00927412

[20] Murray HW. Cellular resistance to protozoal infection. Annu Rev Med. 1986;37:61–9. 10.1146/annurev.me.37.020186.000425 . 3010809

[21] Meinhof W. [Cellular resistance to infection in mycoses]. Z Hautkr. 1979;54(8):322–4. . 442748

[22] McNally BA, Trgovcich J, Maul GG, Liu Y, Zheng P. A role for cytoplasmic PML in cellular resistance to viral infection. PLoS One. 2008;3(5):e2277 10.1371/journal.pone.0002277 18509536 PMC2386554

[23] McGregor DD, Logie PS. The mediator of cellular immunity. VI. Effect of the antimitotic drug vinblastine on the mediator of cellular resistance to infection. J Exp Med. 1973;137(3):660–74. 10.1084/jem.137.3.660 4144033 PMC2139384

[24] McGregor DD, Hahn HH, Mackaness GB. The mediator of cellular immunity. V. Development of cellular resistance to infection in thymectomized irradiated rats. Cell Immunol. 1973;6(2):186–99. 10.1016/0008-8749(73)90021-x . 4632757

[25] Mackaness GB. Cellular resistance to infection. J Exp Med. 1962;116:381–406. 10.1084/jem.116.3.381 25240017

[26] Fauve RM, Delaunay A. [Cellular resistance to bacterial infection. VI. In vitro influence of the ingestion of living or killed bacteria on the bactericidal potency of macrophages towards Listeria monocytogenes]. Ann Inst Pasteur (Paris). 1967;112(4):458–67. . 4965610

[27] el-Assal FM, Shoukry NM, Abdalla MH, Saad AH. Cellular response to Schistosoma mansoni infection in Biomphalaria alexandrina strains selected for susceptibility and resistance. J Egypt Soc Parasitol. 2001;31(3):915–38 + 2p plate. . 11775117

[28] Clomegah AM, Chapman SJ. Resistance to cellular HIV infection. Evol Med Public Health. 2015;2015(1):204 10.1093/emph/eov016 26297685 PMC4547191

[29] Ataev GL, Coustau C. Cellular response to Echinostoma caproni infection in Biomphalaria glabrata strains selected for susceptibility/resistance. Dev Comp Immunol. 1999;23(3):187–98. . 10402206 10.1016/s0145-305x(99)00023-3

[30] Nathan CF, Murray HW, Wiebe ME, Rubin BY. Identification of interferon-gamma as the lymphokine that activates human macrophage oxidative metabolism and antimicrobial activity. J Exp Med. 1983;158(3):670–89. 10.1084/jem.158.3.670 6411853 PMC2187114

[31] Celada A, Gray PW, Rinderknecht E, Schreiber RD. Evidence for a gamma-interferon receptor that regulates macrophage tumoricidal activity. J Exp Med. 1984;160(1):55–74. 10.1084/jem.160.1.55 6330272 PMC2187421

[32] Pace JL, Russell SW, Schreiber RD, Altman A, Katz DH. Macrophage activation: priming activity from a T-cell hybridoma is attributable to interferon-gamma. Proc Natl Acad Sci U S A. 1983;80(12):3782–6. 10.1073/pnas.80.12.3782 6407020 PMC394136

[33] Stein M, Keshav S, Harris N, Gordon S. Interleukin 4 potently enhances murine macrophage mannose receptor activity: a marker of alternative immunologic macrophage activation. J Exp Med. 1992;176(1):287–92. 10.1084/jem.176.1.287 1613462 PMC2119288

[34] Doyle AG, Herbein G, Montaner LJ, Minty AJ, Caput D, Ferrara P, et al Interleukin-13 alters the activation state of murine macrophages in vitro: comparison with interleukin-4 and interferon-gamma. Eur J Immunol. 1994;24(6):1441–5. 10.1002/eji.1830240630 . 7911424

[35] Mills CD, Kincaid K, Alt JM, Heilman MJ, Hill AM. Pillars Article: M-1/M-2 Macrophages and the Th1/Th2 Paradigm. J. Immunol. 2000 164: 6166–6173. J Immunol. 2017;199(7):2194–201. 10.4049/jimmunol.164.12.6166 . 28923981

[36] Mills CD, Kincaid K, Alt JM, Heilman MJ, Hill AM. M-1/M-2 macrophages and the Th1/Th2 paradigm. J Immunol. 2000;164(12):6166–73. 10.4049/jimmunol.164.12.6166 . 10843666

[37] Jenkins SJ, Allen JE. Similarity and diversity in macrophage activation by nematodes, trematodes, and cestodes. J Biomed Biotechnol. 2010;2010:262609 10.1155/2010/262609 20145705 PMC2817371

[38] Bertuzzi A, Fasano A, Gandolfi A, Sinisgalli C. Necrotic core in EMT6/Ro tumour spheroids: Is it caused by an ATP deficit? J Theor Biol. 2010;262(1):142–50. 10.1016/j.jtbi.2009.09.024 . 19781558

[39] Chan WS, Brasseur N, La Madeleine C, van Lier JE. Evidence for different mechanisms of EMT-6 tumor necrosis by photodynamic therapy with disulfonated aluminum phthalocyanine or photofrin: tumor cell survival and blood flow. Anticancer Res. 1996;16(4A):1887–92. . 8712717

[40] Freyer JP, Sutherland RM. Proliferative and clonogenic heterogeneity of cells from EMT6/Ro multicellular spheroids induced by the glucose and oxygen supply. Cancer Res. 1986;46(7):3513–20. . 3708583

[41] Kankaanranta L, Seppala T, Koivunoro H, Saarilahti K, Atula T, Collan J, et al Boron neutron capture therapy in the treatment of locally recurred head and neck cancer. Int J Radiat Oncol Biol Phys. 2007;69(2):475–82. Epub 2007/08/11. S0360-3016(07)00555-X [pii] 10.1016/j.ijrobp.2007.03.039 . 17689034

[42] Trivillin VA, Pozzi ECC, Colombo LL, Thorp SI, Garabalino MA, Monti Hughes A, et al Abscopal effect of boron neutron capture therapy (BNCT): proof of principle in an experimental model of colon cancer. Radiat Environ Biophys. 2017;56(4):365–75. Epub 2017/08/10. 10.1007/s00411-017-0704-7 . 28791476

[43] Yang ZJ, Chee CE, Huang S, Sinicrope F. Autophagy modulation for cancer therapy. Cancer Biol Ther. 2011;11(2):169–76. 10.4161/cbt.11.2.14663 21263212 PMC3230308

[44] Maitz CA, Khan AA, Kueffer PJ, Brockman JD, Dixson J, Jalisatgi SS, et al Validation and Comparison of the Therapeutic Efficacy of Boron Neutron Capture Therapy Mediated By Boron-Rich Liposomes in Multiple Murine Tumor Models. Transl Oncol. 2017;10(4):686–92. 10.1016/j.tranon.2017.05.003 28683435 PMC5498409

[45] Kueffer PJ, Maitz CA, Khan AA, Schuster SA, Shlyakhtina NI, Jalisatgi SS, et al Boron neutron capture therapy demonstrated in mice bearing EMT6 tumors following selective delivery of boron by rationally designed liposomes. Proc Natl Acad Sci U S A. 2013;110(16):6512–7. 10.1073/pnas.1303437110 23536304 PMC3631690

[46] den Bakker MA, Flood SJ, Kliffen M. CD31 staining in epithelioid sarcoma. Virchows Arch. 2003;443(1):93–7. 10.1007/s00428-003-0829-8 . 12743818

[47] Wang D, Stockard CR, Harkins L, Lott P, Salih C, Yuan K, et al Immunohistochemistry in the evaluation of neovascularization in tumor xenografts. Biotech Histochem. 2008;83(3–4):179–89. 10.1080/10520290802451085 18846440 PMC2651088

[48] Sene A, Khan AA, Cox D, Nakamura RE, Santeford A, Kim BM, et al Impaired cholesterol efflux in senescent macrophages promotes age-related macular degeneration. Cell Metab. 2013;17(4):549–61. 10.1016/j.cmet.2013.03.009 23562078 PMC3640261

[49] Chen DL, Engle JT, Griffin EA, Miller JP, Chu W, Zhou D, et al Imaging caspase-3 activation as a marker of apoptosis-targeted treatment response in cancer. Mol Imaging Biol. 2015;17(3):384–93. 10.1007/s11307-014-0802-8 25344147 PMC4874215

